# Modern Socialism

**Published:** 1889-11-02

**Authors:** 


### Modern Socialism.

By a Student of Present-Day Movements.

Socialism is in the air. "We are all Socialists now," said a statesman recently, although it is doubtful if he realised the full meaning of his words, and still more doubtful if any but a small, though active, minority really accept the socialist idea. When public opinion was ruled by the old school of political economy, a dock director might fearlessly have talked, as Mr. Norwood did, of the "economic absurdity" of paying more for labour than the lowest price for which it could be got; public opinion of to-day, moved by the social sentiment that has worked its way into so many minds, declared emphatically that if this lowest price meant semi-starvation for the labourer, an "economic absurdity" must be perpetrated.

"What is socialism?" is a question commonly asked. It might be answered in the saying of St. Paul, "No man liveth to himself, and no man dieth to himself"; in life and in death he is either a boon or a bane to the community, and the community has a right to prevent him being a bane. This rule lies, in intention, at the bottom of all our laws; but it must be admitted that intent and execution differ very widely, and while perverse human nature is constantly varying its methods of ill-doing, it is to be feared that punishment will not always follow on the heels of sin. In other words, the individualist instinct—call it the struggle for existence, the keenness of competition, or any other name you will—is often too powerful and too acute for the social sentiment on which our laws are based. Bill Sykes, in burglariously appropriating the goods another man has bought and paid for, is obeying a pronounced instinct of individualism, and one which the law punishes when it has the chance. But one need not go so far as the much-abused Stock Exchange or the sweater's den to see instances of individualism profiting at the expense of the community, which, as yet, the law cannot touch. How is this to be prevented?

The reply of the Socialist may be formulated in the words of Dr. Albert Schäffle, a former Austrian Minister of Finance*: "To replace the system of private capital (*i.e.*, the speculative method of production, regulated on behalf of society only by the free competition of private enterprises) by a system of collective capital—that is, by a method of production which would introduce a unified (social or 'collective') organisation of unified labour, on the basis of *collective* or common *ownership* of the *means of production* by all the members of the society."

It is necessary to state clearly, as Dr. Schaffle shows in this book which deserves to be widely read, that socialism does not imply a collective ownership in the results produced. Some Socialists protest that it does, and hence comes the popular idea that in a socialist state the industrious must work to support the lazy. Perhaps, in really socialistic conditions that would be less likely to occur than now. Fourrierism, with its collective dormitories and dining-rooms, its absolute negation of personal affections and preferences, could never obtain for long, could hardly gain a footing among a free and energetic people. The weekly equalisation of property which the comic papers describe, and even serious speakers sometimes regard as a fundamental part of socialism, has no real place in the socialist programme, and no one who has a real idea of socialism imagines that it would be for the benefit of the community—which is the end always to be borne in mind—that the loafer and the idler, the gambler and the drunkard, should be as well off as the industrious citizen.

It may be objected that culture and enjoyment will cease in our communal state, for they depend on riches. To a certain extent they do, and it is to be regretted that they are attainable by so few. All the numerous societies and institutions which have been established with the purpose of brightening the lives of the poor are the expression of that regret, and are an attempt to mitigate the action of riches on poverty. For it is idle to pretend that, when only a certain sum of wealth is attainable, the poor can become richer without the rich becoming poorer. But it may be noted that culture and riches do not invariably increase in direct proportion to each other. Below a certain economic point culture and enjoyment are impossible; that is all we dare to say. But we may add that any system which keeps a large proportion of the population below that economic point is bad, and ought to be abolished at any cost, even though the luxuries of the comparatively small number of the rich be cut off in the process.

"Oh! then we shall be all alike!" Not quite. Certain forms of labour, demanding more of training, intellect, and special faculty than others, will still command a higher price when the State rather than the capitalist is the paymaster. The scavenger will not receive the same salary as the artist. It is true that our future scavenger may now be a peer, the inheritor of large estates, and of the prerogative of doing nothing unless he so chooses. It is true that the artist may be fain to give his fancies vent in some dark room after a long day's work is done. The artist might then have better training than he has now; the scavenger would be sure to find his level, when private property in the means of production was abolished.

What does private property in the means of production mean? Land, factories, machines, mines, everything from which men by the sweat of their brows derive the means of living. The State would then be landlord, and what it took in rent would be returned in remission of taxes. It may be surmised, too, that the State would be a less exacting landlord than the private capitalist, who wishes to have the largest possible interest on the money he invests, and feels, indeed, that he is neglecting his duty to his family if he does not do so. It would not put a penny into the pocket of, say the Minister of Agriculture, if a farm brought in ten per cent. on the money the State had given for it. The temptations to rack-renting and to other forms of oppression would be gone. The vulgar idea, however, that the squire's land is to be divided among the labourers on the estate, who thereupon begin quarrelling about the ownership of the best plots, the idea that the master will be abolished, and every factory hand have a carriage and pair—these are as absurd as the notion held on the other side that socialism means barricades, petroleum, and the guillotine. A master and a landlord there will be—the State, impartial, and almost automatic, which would take from the nation to give back to the nation, regulating the distribution of national wealth, but not absorbing it. Socialism is not, as many people think, individualism let loose, and society reduced to a chaotic rivalry of beasts of prey. Neither has it anything to do with "free love" or other wild dreams of individual fanatics. It is the misfortune of any great social movement that it becomes a cave of Adullam for malcontents of every description; but as we do not discredit David because some of his followers were unruly, we need not discredit socialism because of the follies spoken in its name. Doubtless when it comes it will not be free from abuses—what great movement ever was? But, as with slow, unhasting, unresting step it moves along, it is ameliorating the condition of the people—equalising the conditions of existence to the benefit of both rich and poor. Like other kingdoms, it cometh not with observation; but in such modest things as the increasing tendency of municipal bodies in different towns to keep under their own control the lighting, water supply, and means of locomotion, we see the socialistic tendency growing day by day. It offers a high ideal of justice and happiness, and its best friends will hope that it may not suffer from the rash utterances of enthusiastic, but ill-informed, followers.

* "The Quintessence of Socialism." By Dr. A. Schaffle. Translated from the eighth German edition under the supervision of Bernard Bosanquet, M.A. London: Swan Sonnenschein and Co

